# A strategic vision in Neurology

**Published:** 2015

**Authors:** VL Purcarea

**Affiliations:** * “Carol Davila” University of Medicine and Pharmacy, Bucharest, Romania

As it is well known by specialists, stroke represents the most frequent neurological pathology, followed by Alzheimer dementia, and, unfortunately, our country is among the ones with the highest incidence of strokes. At the European level, one citizen out of three suffers from neurological and psychiatric affections, from sleeping disorders to cerebrovascular or neurodegenerative diseases.

In fact, stroke represents the third cause of death in Romania after cardiovascular diseases and cancer. The mortality rate is of 300 cases per one hundred thousand inhabitants in Romania, much higher as compared to the European average level. 

**Fig. 1:**
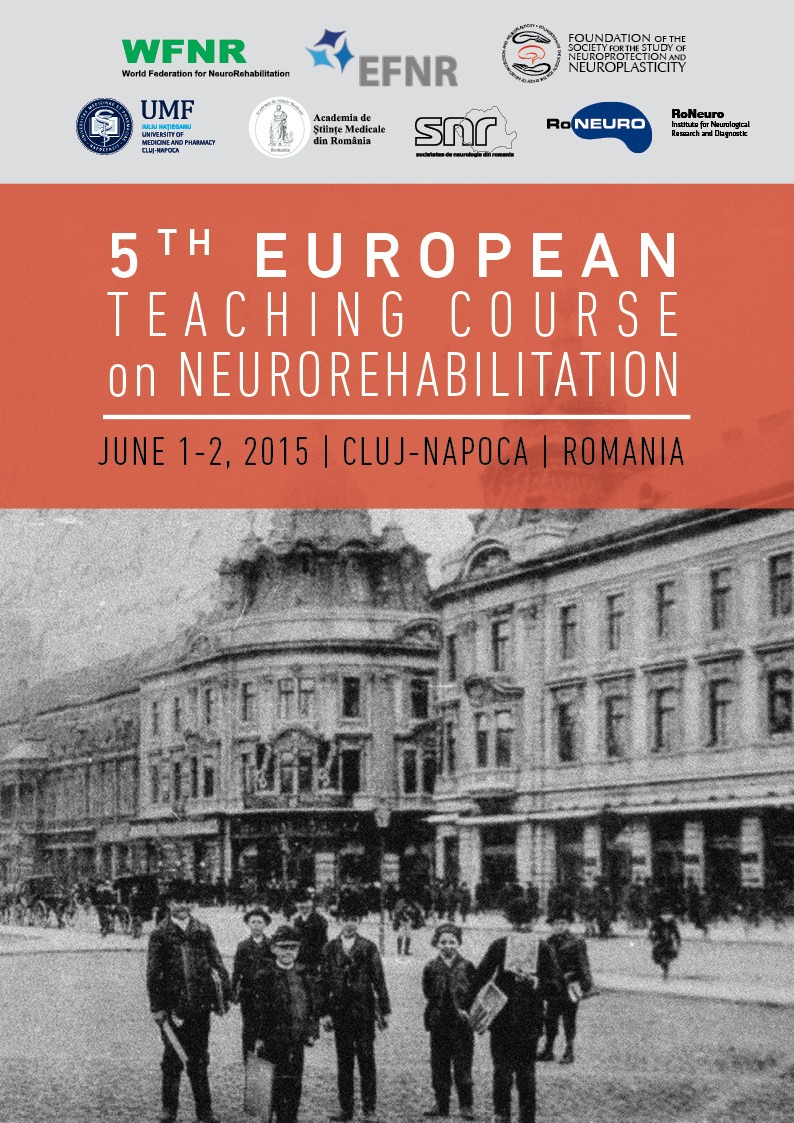
Poster announcing the 5th European Teaching Course on Neurorehabilitation, June 1-2, 2015, Cluj-Napoca, Romania

What seems to be concerning is the fact that this kind of accidents have started to appear, more and more often, around the age category of less than 30 years, so that, at present, as compared to 1990 when the value was of 25%, over 31% of the total number of patients suffering from stroke have ages between 20 and 26 years. Statistics show that from the approximately 65.000 Romanian inhabitants who annually have a stroke, a third remain with diverse degrees of disabilities as a result of a stroke. For the latter, but also for all the people with neurological deficits due to other causes, neurorehabilitation represents a chance of turning back to a life that is as close to normal as possible. As distinguished **Prof. Dafin F. Mureșanu**, MD, President of the Romanian Society of Neurology (RSN) and President of the Society for the Study of Neuroprotection and Neuroplasticity (SSNN) affirmed: ***„Neurorehabilitation represents a complex therapeutic process through which a person with a certain degree of disability, no matter its etiology, can recover the physical, cognitive, emotional and psycho-social abilities, however, limited by the type and complexity of the lesion, as well as the individual biological reserve”***.

The therapeutic strategies in neurorehabilitation have radically changed in the last decades, an integrated approach of both the physical component and the other ones, such as the cognitive and emotional, has been adopted, which generates clinical results that are clearly better than those of the classic one. 

Similar to ecosystems, internet, metabolic pathways, stock market, great urban systems, the human brain is also a complex system. At present, modeling the complex systems represents a world priority of the scientific research. The researches have evidenced that all these complex systems have in common the network type similar structures. The networks theory has become the most visible tool for knowledge, which can be applied in the description, analysis and understanding of the complex systems. 

The unprecedented development of informational technologies has made it possible that every domain targeted in this holistic process should benefit from the progress of the current technologies: robots assisted therapies, new pharmacological and non-pharmacological strategies (magnetic and electric stimulation, etc.) of stimulating neuroplasticity, programs based on the use of “virtual reality” principles, and these are only a few. The complete understanding of the mechanisms of protection and regeneration of the nervous system has allowed the access to a personalized vision of the specific therapies, which can address the disorders of a patient.

The most current results of research in neurorehabilitation, as well as in more advanced therapeutic standards, will be presented in the **5th European Teaching Course on Neurorehabilitation which will take place in Cluj-Napoca, on 1-2 June 2015**. The event, organized by the Foundation of the Society for the Study of Neuroprotection and Neuroplasticity (SSNN), together with the Romanian Society of Neurology (RSN) and “Iuliu Hațieganu” University of Medicine and Pharmacy in Cluj-Napoca, under the auspices of the European Federation of Neurorehabilitation Societies (EFNRS) and the World Federation for Neurorehabilitation (WFNR), will bring in Romania famous people in the field of medicine and scientific research. 

On this occasion, the participants will have the opportunity of visiting the most modern and complex Institute for Neurological Research and Diagnostic in Romania, **“RoNeuro”**, which intends to become an excellence center in Romania and Eastern Europe, dedicated to research and diagnosis of neurological diseases.

**Fig. 2:**
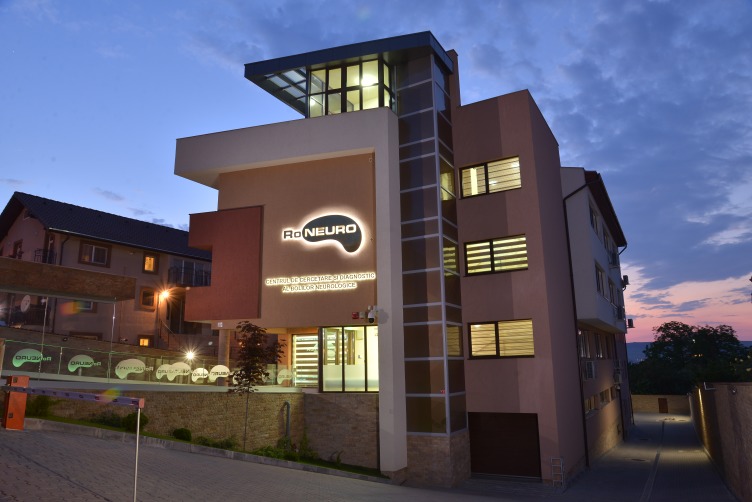
**"RoNeuro”** Institute for Neurological Research and Diagnostic in Romania

***“The medical approach is integrative, mainly dictated by the needs of the patient. We are concerned with the health of the community we live in and we wish to essentially contribute to the development of a healthier society”***, represents the belief of **Prof. Dafin Mureșanu, MD,** who created the concept and directly coordinates the entire medical and research staff of the clinic. 

The whole medical and research activity is built according to rules set by the most important “not for profit” organizations in the United States of America. In addition, as the famous professor stated at the 4th European Teaching Course on Neurorehabilitation: “We wish to develop neurorehabilitation in Romania in a modern, interdisciplinary context, in which the neurologist, the specialist in physical and medical rehabilitation, the psychologist, the logopedist, the kinesiotherapist, the ergotherapist and the nurse, would work in a team and generate the optimum result for the patient. ***“In the near future, we will also try to bring a competency in the operational area, in the field of neurorehabilitation, a curriculum already being developed at the European level. Neurorehabilitation is an interdisciplinary field currently developing in Europe. Many national societies are born under our watch; of course, the leaders in the field are still the national societies in Germany, Italy, Netherlands, and Austria. Romania is also active in this field, thanks to the two societies of neurorehabilitation”***. 

The knowledge and creativity are key factors of the current society, which is full of opportunities, a society in which globalization and liberalization have generated a dynamic competitiveness, in which the focus is on the ability to innovate, on the receptivity to the health needs and not on the relationship between costs and benefits. Even if it is easier to obtain, the information in the medical field is harder to manage. In an economy in which the fight for acquiring the information, realizing and owning knowledge is of vital importance, innovation, technology and dynamism are the key levers of progress and the rethinking of the communication behavior with the patient has become a must.

That is why this new and useful **European Teaching Course on Neurorehabilitation, which will take place in Cluj-Napoca**, obviously represents a very clear concept of strategic vision, focused on the anticipation of transformations and interactions, an essential condition for any participant, system or health organization which is mature enough and has only one main denominator, “the quality of life”. 

**Executive Editor** **Assoc. Prof. Dr. Eng. ** **Victor Purcarea**

